# Estimating the spatial distribution of wintering little brown bat populations in the eastern United States

**DOI:** 10.1002/ece3.1215

**Published:** 2014-09-08

**Authors:** Robin E Russell, Karl Tinsley, Richard A Erickson, Wayne E Thogmartin, Jennifer Szymanski

**Affiliations:** 1 U.S. Geological Survey, National Wildlife Health CenterMadison, Wisconsin; 2 Division of Endangered Species, U.S. Fish and Wildlife ServiceBloomington, Minnesota; 3 U.S. Geological Survey, Upper Midwest Environmental Sciences CenterLa Crosse, Wisconsin; 4 Division of Endangered Species, U.S. Fish and Wildlife ServiceOnalaska, Wisconsin

**Keywords:** Bats, decision-making, *Myotis lucifugus*, spatial modeling, species distribution modeling, white-nose syndrome

## Abstract

Depicting the spatial distribution of wildlife species is an important first step in developing management and conservation programs for particular species. Accurate representation of a species distribution is important for predicting the effects of climate change, land-use change, management activities, disease, and other landscape-level processes on wildlife populations. We developed models to estimate the spatial distribution of little brown bat (*Myotis lucifugus*) wintering populations in the United States east of the 100th meridian, based on known hibernacula locations. From this data, we developed several scenarios of wintering population counts per county that incorporated uncertainty in the spatial distribution of the hibernacula as well as uncertainty in the size of the current little brown bat population. We assessed the variability in our results resulting from effects of uncertainty. Despite considerable uncertainty in the known locations of overwintering little brown bats in the eastern United States, we believe that models accurately depicting the effects of the uncertainty are useful for making management decisions as these models are a coherent organization of the best available information.

## Introduction

Management and conservation of wildlife species requires accurate information on the spatial distribution of a species in order to identify habitats important for sustaining populations (Thogmartin and Knutson 2007; Guisan et al. 2013). However, obtaining accurate information on spatial distributions of wildlife species is often difficult particularly for species such as bats that range widely across a variety of habitats (Kunz and Fenton 2003; Altringham 2011). In general, the process of mapping a species distribution requires knowledge of the ecological process generating the observed distribution, a statistical model that includes important predictor variables and determines the structure of the estimated error, and a data stream, usually consisting of records of species presence and/or absence (Austin 2002; Fitzgerald et al. 2008; Elith and Leathwick 2009). Species distribution models (SDM), like all models, include varying amounts of error including deficiencies in the observations, such as misidentification of species, inaccurate geographic locations, lack of information on true absences, and a lack of knowledge regarding the ecological process that is generating the observations (Barry and Elith 2006; Laurent et al. 2010). By accurately portraying uncertainty in the state of knowledge surrounding the predicted population distribution of wildlife, decision makers can proceed with an informed decision that accounts for this uncertainty (Harwood and Stokes 2003). However, the quantification and portrayal of uncertainty in species distribution modeling is often neglected.

Estimating the spatial distribution of the wintering population of bats is particularly subject to uncertainty because of the difficulty in conducting systematic surveys for overwintering sites and estimating the size of the overwintering population. Without systematic surveys, our ability to estimate the number of undetected but occupied hibernacula is limited because no quantitative estimates of detection probabilities exist. As recently as 2012, a new hibernaculum containing tens of thousands of bats, including the endangered Indiana myotis bat (*Myotis sodalis*), was discovered in Missouri (USFWS 2013), indicating that our current knowledge of hibernacula is incomplete. Once discovered, the size of the bat population inside the hibernaculum is also difficult to estimate. Observers are limited by time, because staying in the cave for long periods rouses the bats (Kunz et al. 1996), and are constrained to accessible parts of the cave, often leaving areas deeper in the cave unsurveyed. Additionally, bats in hibernacula are often packed close together and/or overlapping each other, making visual counts difficult (Kunz et al. 1996). Lastly, identifying individual species in mixed species hibernacula can be difficult.

Currently, bat populations are experiencing population-level changes from a variety of threats including white-nose syndrome (WNS) (Frick et al. 2010; Dzal et al. 2011; and Foley et al. 2011) and wind energy development (Kunz et al. 2007; Arnett et al. 2008; Arnett and Baerwald 2013). Declines in bat populations not only represent a loss of biodiversity but also affect the ecosystem services bats provide (López-Hoffman et al. 2014). For example, insectivorous bats provide natural pest control by consuming large volumes of insects, seed- and pollen-eating bats assist with plant reproduction by dispersing seeds and pollen, and bats redistribute nutrients through the production of guano (Kunz et al. 2011). The economic loss of ecosystem services from reductions in bat populations caused by WNS has been estimated at $3.7 billion per year (Boyles et al. 2011). Despite these important services, much remains unknown regarding bat populations, including estimates of bat abundance, distributions of bat species, and locations of overwintering hibernacula.

Little brown bats (*M. lucifugus*) are cave-dwelling bats that are distributed widely throughout the United States. In the eastern United States, little brown bats primarily overwinter in caves and mines (Fenton and Barclay 1980) unlike other species of bats such as big brown (*Eptesicus fuscus*) (Whitaker and Gummer 1992). White-nose syndrome is a disease of cave-dwelling bats caused by the fungus *Pseudogymnoascus destructans* (Blehert et al. 2009) that was first discovered in 2006 in a cave in upstate New York (Hefferman 2014). Surveillance indicates the disease has reached as far west as Missouri, as far south as central South Carolina and northern Alabama, and as far north as the Canadian Provinces of Nova Scotia, New Brunswick, Ontario and Quebec (Hefferman 2014).

Research indicates little brown bats are particularly susceptible to the effects of WNS, likely due to their propensity for overwintering colonially, and populations in the northeastern United States have undergone recent declines attributed to the disease (Frick et al. 2010; Brooks 2011; Dzal et al. 2011). Precise estimates of little brown bat population sizes are nonexistent; however, Frick et al. (2010) estimated little brown bat populations at 6.5 million pre-WNS with an average of 75% population declines at affected hibernacula. To assess the current status of the little brown bats in light of WNS and other environmental stressors, we developed a model of little brown bat wintering population size and spatial distribution that captures the uncertainty in our current state of knowledge.

We used information solicited from state agencies on known hibernacula locations to develop models estimating the number of little brown bat hibernacula per county for the United States east of the 100th meridian. We quantified the uncertainty associated with this process by simulating multiple realizations of the model assuming different population sizes and assessing the variability between the outcomes. Output from this model can be used as a basis for developing conservation and management strategies for little brown bats, and the modeling process, we describe is applicable to a wide variety of species with sparse information available on their current distributions.

## Methods

### Data source and parameterization

We conducted an email survey of state biologists located east of the 100th meridian in the United States, for information on known wintering locations of little brown bats. U.S. state agency biologists were requested to provide information on the location of known hibernacula, the number of bats wintering in the hibernacula, and whether WNS had been documented in the hibernacula. States returned information on all hibernacula where little brown bats had been recorded historically, including those where WNS had severely reduced or eliminated bats. Nonresponses or uncertainty in the answers from the email surveys led to follow-up telephone surveys.

We considered biologist estimates of the number of hibernacula per county as minimum counts. We used the estimated counts by county to develop a predictive model of hibernacula counts using spatial and environmental covariates. Specifically, we modeled the observed counts as Poisson random variables *y*_*i*_ ˜ Pois(*λ*_*i*_), where y was the observed counts. Counts were modeled as a function of covariates including the percentage of karst coverage (Tobin and Weary 2004), forest coverage (Xian et al. 2009), an offset term log(A), where A was the area of the county, and Latitudinal and Longitudinal coordinates of the county centroids. Modeling was conducted in a Bayesian framework with WinBUGS (Lunn et al. 2000) and R2WinBUGS (Sturtz et al. 2005). Priors for the intercept term, *α*, and regression coefficients, *β*, were normally distributed random variables; *N*(*μ*,*τ*), with mean (*μ*) of 0 and precision (*τ*) of 0.001 (Note, *τ *= 1/*σ*^2^, where *σ* is the variance). The response variable was related to the covariates with the equation: log(*λ*) = *α *+ log(A) + *β*X, where X was a matrix of covariate values, *β* was a vector of covariate parameters, and *α* was the intercept. We ran 4 candidate models: an intercept-only model (a model of homogeneous spatial distribution), a model with percentage of forest and karst per county, a model with latitude and longitude, and a model with both spatial and environmental variables. We used an information-theoretic approach to model selection using DIC (Spiegelhalter et al. 2002) to select the best model (lowest DIC score = the best fit); this best model provided us with an estimate of the number of hibernacula per county that was used in subsequent steps.

### Model predictions and uncertainty quantification

Once we generated estimates of hibernacula counts per county, we then estimated the size of each hibernaculum using a power-law scaling function developed for Indiana bats. Thogmartin and McKann (2014) described a power-law relation between the frequency of wintering populations, *f*(*n*), for the congeneric Indiana bat (*M. sodalis*) and the size, *n*, of those wintering populations. This relationship was *f*(*n*) ≈ *n*^−0.44^. When the logarithm of this function was calculated, the scaling exponent was coincident with a metabolically defined ¾-power law (West et al. 1997; Brown et al. 2004; Sibly et al. 2012). We assumed a similar power law applied to the size-frequency distribution of little brown bats for determining the number of wintering populations of various sizes. We used a priori information that Tennessee and Kentucky contained smaller hibernacula than northern states within the little brown bat distribution, by drawing random estimates from the distribution of hibernacula sizes below 35,000 bats. This process was simulated 1000 times to generate estimates of the mean number of bats in each county, and provided estimates of little brown bat populations pre-WNS.

Finally, to generate estimates of current bat populations (post-WNS), we relied on expert judgment to develop estimates of the current little brown bat population size and the relevant quantiles (Burgman 2005, Kuhnert et al. 2010). Using standard elicitation protocols (Kahneman et al. 1982, Morgan and Henrion 1990; O'Hagan et al. 2006), we requested that 16 species experts provide an answer to the following question, “What is your best estimate of the current annual little brown bat population size for the continental US east of the 100th meridian?” (see Szymanski 2013 for details on the expert solicitation process). Additionally, participants were provided with a map and asked to designate “a rough boundary that contains 80% of the current (2012) eastern little brown bat population” (taking the effects of WNS into account). Experts were invited to review and discuss their responses with other participants prior to recording their final opinions.

The final core area was determined by taking the average of the expert's estimates of the boundary lines drawn in response to the above question. On the basis of the experts' opinions, we separated counties into two categories, “within the core area” of current little brown bat populations and “outside the core area”. We randomly reordered the counties, and then selected the counties according to their randomized order until the target population size (the target population size is either the mean, median, lower or upper quantile of the expert's estimates) was reached. For the “within core area”, we stopped selecting counties when we reached 80% of the target population size. For the category “outside the core area”, we stopped selecting counties when we reached 20% of target population size. We repeated this process 1000 times for each of 4 expert-estimated population sizes (mean, median, upper and lower quantiles from expert opinions).

## Results

### Hibernacula information

We received information on 1788 hibernacula located in 18 states: Arkansas, Connecticut, Iowa, Illinois, Indiana, Kentucky, Massachusetts, Maryland, Maine, Michigan, Minnesota, New Jersey, New York, Ohio, Tennessee, Virginia, Vermont, and Wisconsin. No little brown bat hibernacula were reported for Alabama, Delaware, Florida, Georgia, Kansas, Louisiana, and Mississippi. Information on hibernacula was provided in various forms by different states including estimates by county, estimates for the state, or general descriptions of the distribution (i.e., ˜100 hibernacula in the northwest section of the state).

**Figure 1:**
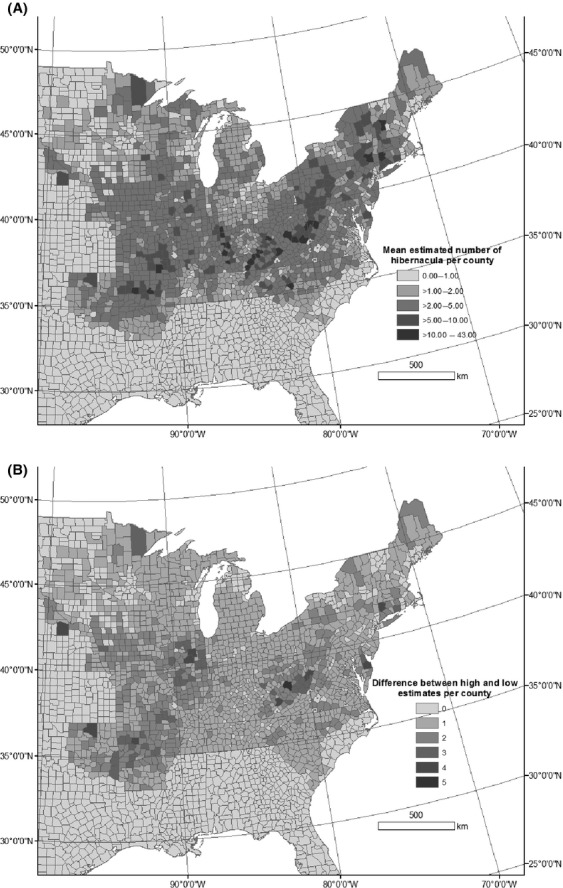
(A) Estimated number of hibernacula per county for the mean population size, (B) difference between the estimated number of hibernacula for the upper and lower quartile populations sizes.

The best model of hibernacula counts was the full model with percent forest, percent karst, and linear and quadratic relationships between latitude and longitude. Parameter estimates indicated that the percentage of forest in a county was positively related to the number of hibernacula (Table1), while the percentage of karst in a county had an ambiguous relationship with the number of hibernacula (i.e., the credible interval of the parameter estimate included zero). There was also a spatial trend indicating increasing numbers of hibernacula in the eastern versus western portion of the range (a positive parameter estimate for latitude) and a quadratic trend in longitude with a peak in the center of the north–south range. The mean estimated number of hibernacula from the Poisson model was 4061 (median = 4053, [95% Credible Interval 3616–4539]) (Fig.1A). The average coefficient of variation by county for the estimated number of hibernacula was ˜13%. The states with the highest number of estimated hibernacula were Kentucky, Missouri, and Illinois. The sum of the total number of hibernacula estimated for each county assuming a mean population estimate was 444, 345, and 301 for Kentucky, Missouri, and Illinois, respectively. The states with the largest difference between the number of hibernacula estimates given the population size was 6.5 million (the upper quantile) versus 2.5 million (the lower quantile) were Missouri, Illinois, and Arkansas, with a difference of 170, 155, and 136, respectively (Fig.1B).

**Table 1:** Mean, standard deviation, lower credible interval (LCL), upper credible interval (UCL), and 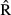 (a measure of fit) for standardized parameter estimates from Poisson models of hibernacula counts by county.

Parameters	Mean	SD	LCL	Median	UCL	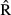
Forest	1.56	0.27	1.03	1.55	2.09	1.00
Karst	−0.16	0.14	−0.43	−0.16	0.11	1.00
Longitude	0.21	0.04	0.12	0.21	0.29	1.00
Latitude	0.16	0.06	0.05	0.16	0.28	1.00
Longitude × Longitude	0.06	0.04	−0.03	0.06	0.14	1.00
Latitude × Latitude	−0.40	0.05	−0.50	−0.40	−0.30	1.00
Intercept	−6.74	0.19	−7.13	−6.74	−6.38	1.00

**Figure 2:**
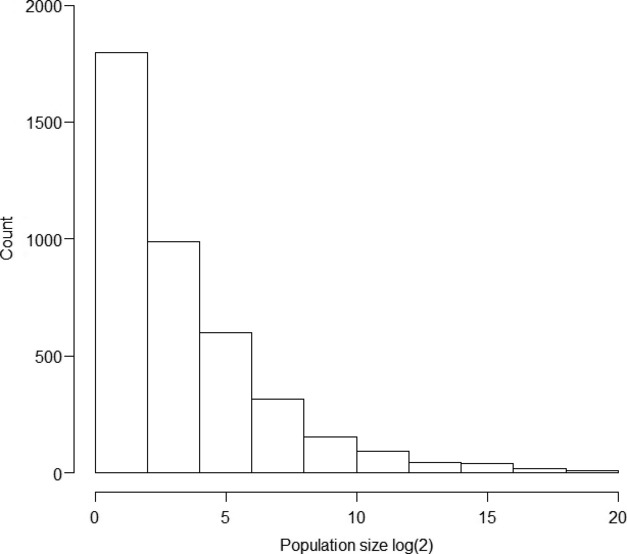
Distribution of hibernacula sizes on the log2 scale for 1000 random draws for the estimated 4061 hibernacula.

**Figure 3:**
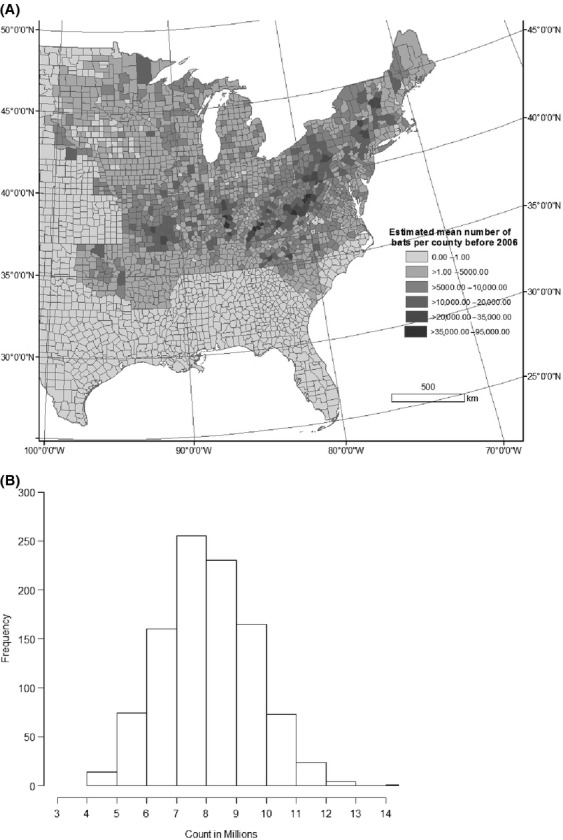
(A) Estimated population size pre-WNS based on mean estimated number of hibernacula. (B) Distribution of the estimated population size of little brown bats in the eastern United States, pre-WNS.

**Figure 4:**
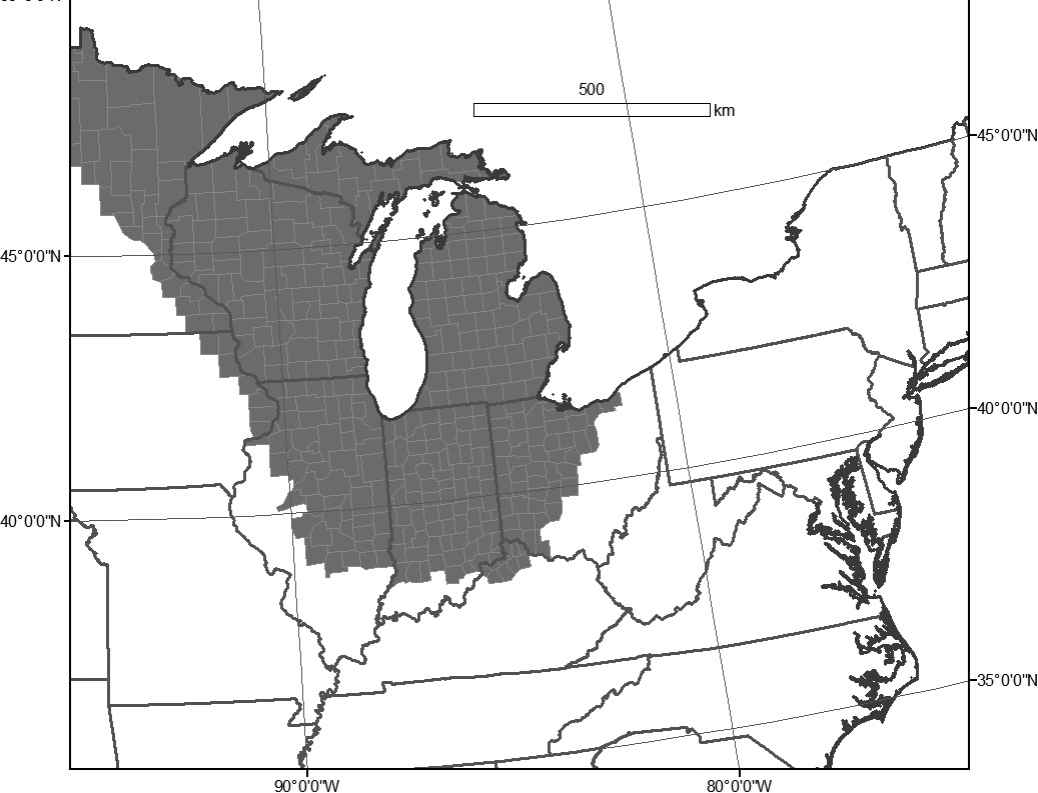
Geographical distribution of the current little brown bat population; gray areas are estimated to contain 80% of the population in 2013.

Results from 1000 different draws for the 4061 hibernacula from the size distribution of hibernacula (Fig.2) indicated a mean estimated total population of bats pre-WNS of 8,136,854 bats (median = 7,991,403, [95% Credible Interval 5,450,737–11,127,397]) (Fig3A and B). The average coefficient of variation per county in the estimated number of bats was ˜110%. For the current distribution of little brown bats, 11 species experts provided estimates of current population size and population distribution of little brown bats in the eastern United States. The mean population size estimated by the experts was 5.5 million surviving little brown bats in 2012 (median = 4 million, 25th percentile = 2.5 million, 75th percentile = 6 million). The experts estimated the core population (where 80% of the population remained) of current little brown bats to be in the upper Midwest region (Fig.4). Hibernacula count estimates per county were made under different assumptions of population size (4 million, 5.5 million, 2.5 million, and 6 million). The assumed population size resulted in considerable within scenario variation in the estimated number of hibernacula and estimated number of bats in each county (Table2).

**Table 2:** Estimated mean number of hibernacula for estimated total population sizes of 2.5 million, 4 million, 5.5 million, and 6 million. Mean CV is the mean coefficient of variation in the number of hibernacula and individual bats across counties for a particular scenario; 95% C.I. indicates the empirical 95% confidence interval obtained from generating 1000 realizations of each estimated population size.

Population Size	Mean Number of Hibernacula	Mean CV Hibernacula	Mean CV Bat number
2.5 Million	1829 [95% C.I. 1084–2474]	96%	1095%
4 Million	2209 [95% C.I. 1701–2829]	75%	1033%
5.5 Million	2416 [95% C.I. 1841–3171]	63%	978%
6 Million	2470 [95% C.I. 1855–3209]	59%	970%

## Discussion

When making decisions regarding wildlife management and conservation, baseline information regarding “how many” and “where are they” is often lacking for wildlife species (Guisan et al. 2013). Obtaining accurate estimates of the spatial distribution of a wildlife species is often not possible in the face of rapidly changing environmental conditions resulting from disease, fire, human development, or other large-scale disturbances. Species distribution models are essential for providing guidance regarding the abundance and location of wildlife, however, overconfidence in the results of these models can lead to erroneous decisions. Accurate representation of uncertainty is necessary to provide policy makers with a broader understanding of the current state of knowledge. By providing a description of the uncertainty associated with the prediction process, decision makers can quantify how robust their decisions are in the face of uncertainty (Regan et al. 2005).

In our system, our estimates included model uncertainty, uncertainty in the estimated number of hibernacula per county, uncertainty in the number of bats per hibernacula, and uncertainty in the current total population size. Our best models indicated that increased forest cover was related to increased numbers of hibernacula, an eastern and central distribution of hibernacula, and a negative relationship with percent karst (although the credible interval included zero). We believe the associations we observed are due to the strong correlation between karst and forest (72%), with the effects of forest cover mediating the effects of karst. However, it is possible due to the fact that little brown bats will also overwinter in mines, that percentage of karst in a county as not as strong a predictor as we expected.

Our observed hibernacula data consisted of minimum known hibernacula counts per county with no estimates of detection probabilities. Without estimates of the survey effort that led to these observations, it is difficult if not impossible to quantify the number of hibernacula potentially missed (Thogmartin and McKann 2014). Zeros in our data may indicate that there truly are no hibernacula in a county or that no effort has been expended in identifying hibernacula in that county (Lobo et al. 2010); therefore, our efforts are reduced to presence-only modeling of hibernaculum occurrence. Recent literature has questioned the broadscale use of “presence-only” methodology due to frequent violation of assumptions, including assumptions that observed presences are the consequence of random or representative sampling and that detectability during sampling does not vary with the covariates that determine occurrence probability (Yackulic et al. 2013). Without designed systematic studies to identify new hibernacula and true absences the real spatial distribution of bats remains difficult to estimate.

Individual bats are also notoriously difficult to count and obtaining accurate estimates of overwintering population sizes is problematic (Thogmartin et al. 2012). Our study focused on little brown bats east of the 100th meridian because information regarding little brown bats numbers, and hibernacula distribution west of this line was sparse. Many of these western states reported having large numbers of little brown bats that were distributed widely, but information regarding overwintering habitat locations and the estimated number of bats was largely absent. New methodology using cameras and quantifying survey effort may lead to improvements in count data, but it is not realistic to expect that a thorough census of bat populations rangewide can be achieved without a considerable investment of resources. Despite this, wildlife policy makers must make decisions regarding the effects of alternative energy development, WNS, and other landscape-level processes on bats, therefore, the models and maps we provide should prove to be essential for effective conservation decision-making.

Assessing the effect of the uncertainty in the modeled estimates relative to the management decision at hand is also important. For example, Frick et al. (2010) predicted the extirpation of little brown bats from WNS-affected areas using a nonspatially explicit model that did not capture the uncertainty in the known population size of the species. In the face of this fast-moving disease that imposes severe mortality rates on infected bats, the uncertainty in the number and spatial location of bats in the eastern part of their range may not lead to different conclusions regarding the overall consequences of the disease. However, for predictions of the impact of wind energy development on bat populations, the locations of hibernacula in conjunction with maternity roosts is vital for estimating the amount of mortality that can be expected from new wind farms, although smaller resolution data may be needed. Assessing the collective impacts of these factors on bat populations will be important for providing management guidelines for energy development and other human-mediated landscape-level changes.

## Conclusion

Uncertainty in model parameters should not prevent informed predictions regarding population dynamics of a population in response to a new stressor such as an emerging disease or changing land-use patterns. Uncertainty is inherent in any modeling process, but by identifying parameters contributing most to uncertainty, future studies can focus on reducing uncertainty in parameters most crucial to making improved management decisions (Runge et al. 2011). Models can provide information to managers regarding which actions are most likely to influence the overall impact of the threat on a species. Even with our predicted uncertainty, management recommendations can still be developed. For example, to minimize the impacts of wind energy on bat populations, land managers may want to avoid placing large wind farms in areas with many hibernacula. Little brown bats are philopatric (Norquay et al. 2013) and will likely return to the same hibernacula even if conditions in the cave have deteriorated. Overall minimizing disturbance to overwintering sites is likely crucial for providing little brown bats with their best opportunity for recovery from large-scale landscape-level changes.
